# Multi-omics analysis reveals neoantigen-independent immune cell infiltration in copy-number driven cancers

**DOI:** 10.1038/s41467-018-03730-x

**Published:** 2018-04-03

**Authors:** Daniel J. McGrail, Lorenzo Federico, Yongsheng Li, Hui Dai, Yiling Lu, Gordon B. Mills, Song Yi, Shiaw-Yih Lin, Nidhi Sahni

**Affiliations:** 1 Department of Systems Biology, The University of Texas MD Anderson Cancer Center, Houston, TX 77030 USA; 2 Department of Melanoma Medical Oncology, The University of Texas MD Anderson Cancer Center, Houston, TX 77054 USA; 3 Department of Oncology, Livestrong Cancer Institutes, Dell Medical School, The University of Texas at Austin, Austin, TX 78712 USA; 4 Program in Quantitative and Computational Biosciences, Baylor College of Medicine, Houston, TX 77030 USA; 5 Department of Bioinformatics and Computational Biology, The University of Texas MD Anderson Cancer Center, Houston, TX 77030 USA

## Abstract

To realize the full potential of immunotherapy, it is critical to understand the drivers of tumor infiltration by immune cells. Previous studies have linked immune infiltration with tumor neoantigen levels, but the broad applicability of this concept remains unknown. Here, we find that while this observation is true across cancers characterized by recurrent mutations, it does not hold for cancers driven by recurrent copy number alterations, such as breast and pancreatic tumors. To understand immune invasion in these cancers, we developed an integrative multi-omics framework, identifying the DNA damage response protein ATM as a driver of cytokine production leading to increased immune infiltration. This prediction was validated in numerous orthogonal datasets, as well as experimentally in vitro and in vivo by cytokine release and immune cell migration. These findings demonstrate diverse drivers of immune cell infiltration across cancer lineages and may facilitate the clinical adaption of immunotherapies across diverse malignancies.

## Abstract (web summary)

Neoantigen load has been associated with tumour immune infiltration. Here, the authors show that while this is true for tumours with recurrent mutations, cancers with recurrent CNAs show neoantigen-independent infiltration driven by cytokine production downstream of the DNA damage sensor ATM.

## Introduction

Immune checkpoint inhibitors, such as antibodies targeting PD-L1 and CTLA4, are emerging as a promising new paradigm in cancer treatment. However, a critical gap lies in identifying which patients will respond to these therapies. Current literature largely supports the idea that large mutational burdens will generate neoantigens for T-cell recognition, in turn leading to increased recruitment of CD8^+^ cytotoxic T-cells^1^ which are necessary for efficacy of immune checkpoint blockade^2–4^. Indeed, mutational burden has been shown to correspond to efficacy of checkpoint blockade in melanoma, lung cancer, and colorectal cancer^3,5–7^. This accumulating evidence has resulted in attempts to utilize microsatellite instability, a biomarker for defects in mismatch repair leading to a hyper-mutator phenotype, as a pan-cancer predictive marker for immunotherapy^8^.

However, it remains unclear how broadly informative mutational load is within other cancer lineages. While most current clinical trials are focused on the aforementioned cancers, they are rapidly expanding to more diverse cancers with little knowledge of biomarker conservation. Checkpoint inhibitors targeting both PD1 and CTLA4 are currently underway for hepatocellular carcinoma, triple negative breast cancer, head and neck cancer, and thyroid cancer amongst other malignancies^9^. Recent analysis of genomic correlates of immunotherapy response in clear cell renal carcinoma found mutational burden to have no prognostic value^10^. As these trials continue, there exists a clear need to determine whether mutational burden is a sufficient biomarker across all cancers, and if not what biomarkers could be useful in their stead.

To investigate this, we leveraged multi-omics analysis across 19 different cancer lineages to better understand determinants of CD8^+^ cytotoxic T lymphocyte (CTL) levels. We found only 26% (5/19) of cancer lineages display a significant positive correlation between neoantigen levels and CTLs, corresponding to M-class cancers characterized by frequent mutational drivers. In contrast, C-class cancers driven by recurrent copy number alterations, including those of the breast, pancreas, and bladder showed no relationship. Mutli-omics network analysis identified phosphorylation of the DNA double-strand break signal transducer ATM as a strong predictor of CTL infiltration that may act by driving expression of key cytokines. These predictions were validated using in vitro and in vivo experimental models. Taken together, this work documents diverse determinants of CTL levels across different cancer types and identifies ATM as a potential novel driver of CTL infiltration.

## Results

### Neoantigen load is not a pan-cancer marker of CTL levels

**Fig. 1:**
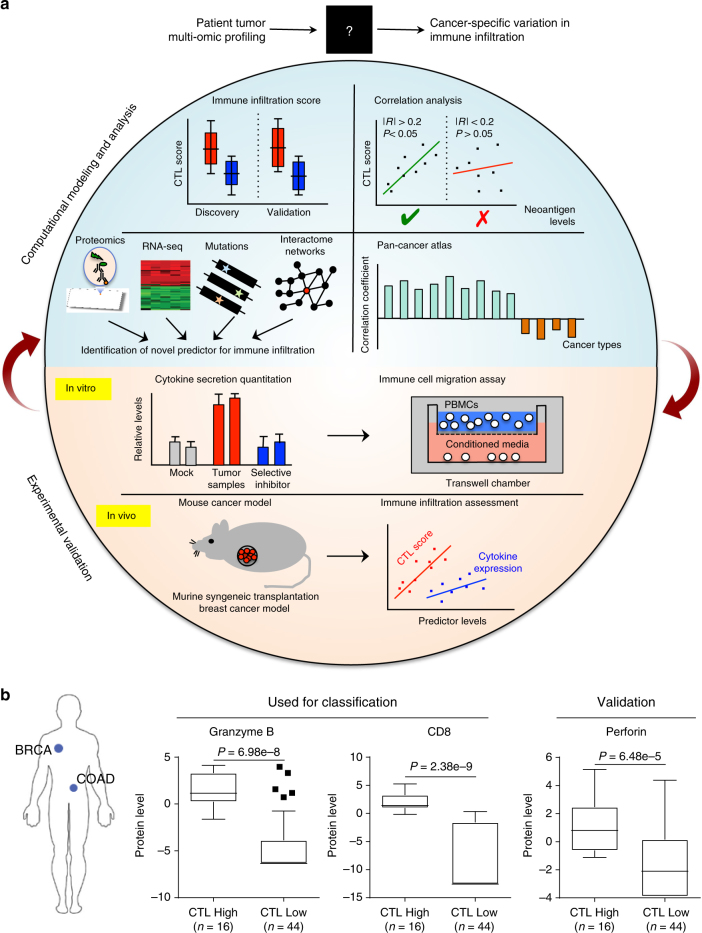
Integrative multi-omics network analysis framework to reveal determinants of immune infiltration across human cancers. **a** Flow chart for analysis of pan-cancer indicators of immune invasion. Cytotoxic T-cell (CTL) levels were determined from proteomic data and integrated with gene expression, genetic mutations, phospho-/total- proteomics, and interactome networks. Identified markers were then expanded pan-cancer, and potential novel drivers were experimentally verified. **b** Protein data sets and classification of CTL high patients. CTL high breast cancer patients were classified by positive expression of the cytolytic enzyme granzyme B and CD8 CTL surface marker, and also showed enrichment of the cytolytic enzyme perforin. *P* values were determined by Wilcoxon rank-sum test. Box indicates median with interquartile range, and whisker length determined by the Tukey’s method

**Fig. 2:**
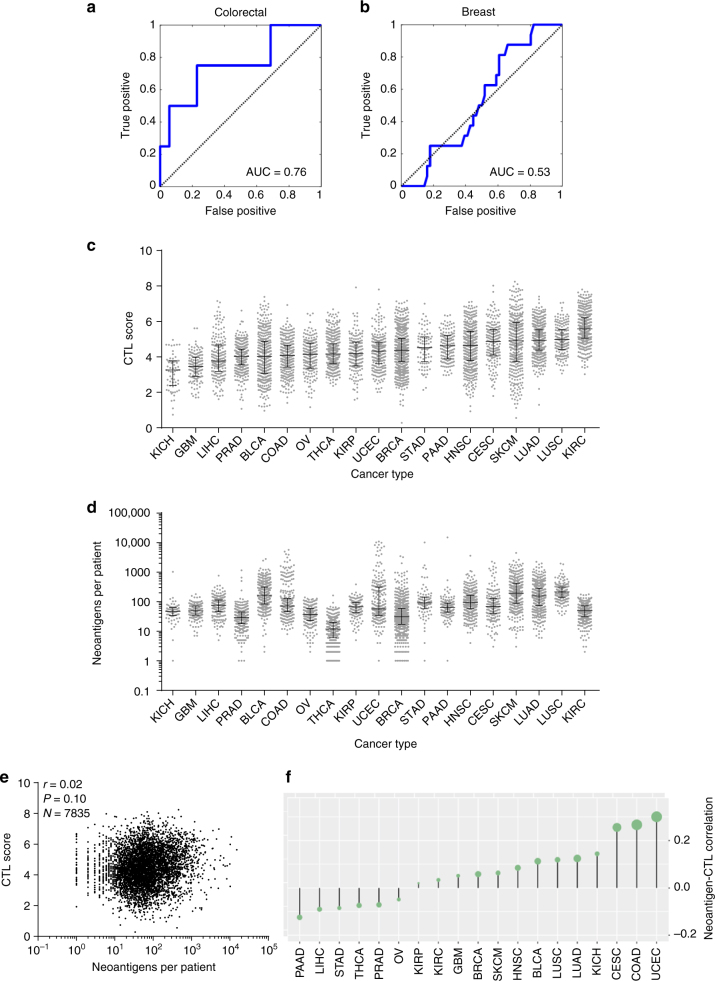
Proteomic identification of patients with high CTL invasion reveals that breast cancer invasion does not correlate with neoantigens. **a** Receiver-operator characteristic plot for prediction of CTL high patients based on neoantigen levels in colorectal cancer patients. Area under the curve (AUC) equal to 0.757 (*N* = 64). **b** Receiver-operator characteristic plot for prediction of CTL high patients based on neoantigen levels in breast cancer patients. Area under the curve (AUC) equal to 0.532 (*N* = 80). **c** CTL score calculated across 19 cancers, plotted in order of median for each cancer. Dots represent individual patients, and lines are median with interquartile range. See Supplementary Fig. 3. Sample sizes given in Supplementary Table 1. **d** Neoantigens per patient across 19 cancers, plotted in order of median CTL score for each cancer. Dots represent individual patients, and lines are median with interquartile range. Sample sizes given in Supplementary Table 1. **e** Plot of CTL score as a function of neoantigen load across all patients (*N* = 7835). Inset *r* is Spearman’s correlation coefficient and corresponding *P* value. **f** Pan-cancer analysis of neoantigen-CTL Spearman correlation, with size of dots topping each bar representing significance level (P). Sample sizes and exact *P*-values given in Supplementary Table 2

To gain a better understanding of CTL infiltration in cancer, we sought to integrate recently published whole-proteome data sets from breast^11^ and colorectal cancer^12^ patients with the abundance of other omic data sets available for these cancers (Fig. 1a). We classified patients as CTL high based on presence of both the marker CD8 and the cytolytic enzyme granzyme B, and validated this classification based on expression of a second cytolytic enzyme perforin (Fig. 1b). As expected for colorectal cancer, CTLs were enriched in microsatellite instable patients (*P* = 1.3 × 10^−3^, Supplementary Fig. 1A) who have a large mutational load. Likewise consistent with previous observations, neoantigen load was strongly related with CTL levels as shown by the receiver-operator characteristic curve for predicting CTL high patients based on neoantigen burden (Fig. 2a). When analyzing breast cancer, we were surprised to find that CTL high patients were distributed throughout all four subtypes with no significant enrichments (Supplementary Fig. 1B). Moreover, the previously observed predictive power of neoantigen load was lost in breast cancer patients, showing no relationship to CTL content (Fig. 2b). Likewise, no enrichment for patients with high copy number variation or overall mutational load was observed (Supplementary Fig. 2).

To investigate this phenomenon across a broader panel of cancers, we trained an RNAseq-based CTL score on this protein data with markers previously shown to be indicative of cytolytic CD8^+^ T-cells^13^ using elastic net regression on the breast cancer patient data. To test the efficacy of the score, we first identified TCGA breast cancer patients with high infiltrating lymphocyte levels based on evaluation of tumor slides by trained histopathologists and found patients with high lymphocytes levels show significantly higher CTL scores than those with low lymphocyte infiltration (*P* = 3.86 × 10^−5^), though CTLs as evaluated by CIBERSORT failed to reach statistical significance when performing the same analysis (*P* = 0.11) (Supplementary Fig. 3A). Furthermore, our CTL score demonstrated strong enrichment in known immuno-oncology biomarkers such as PDL1 protein expression and MSI status (Supplementary Fig. 3B–C), and compared favorably with a panel of other predictive RNAseq approaches (Supplementary Fig. 3D). Finally, we immunostained breast cancer tissue sections from a murine-derived syngeneic transplant model^14^ for the CTL marker CD8 and found the immunostaining data was in good agreement with our RNAseq CTL score (Supplementary Fig. 3E). This score predicts high levels of CTLs in lung and skin cancers, with minimal immune levels in glioblastoma (Fig. 2c). When analyzing neoantigen levels in the same panel of cancers we observed some discrepancies, such as the kidney renal clear cell (KIRC) cohort showing the highest CTL score without significant neoantigen levels (Fig. 2d). To test if neoantigen load was broadly predictive of CTL score, we compared these two parameters across over 7500 patients from these cohorts, finding no significant correlation between the two parameters (Spearman’s *R* = 0.02, *P* = 0.10, Fig. 2e). Finally, we calculated the neoantigen-CTL correlation on a per-cancer basis, finding five cancer cohorts that showed significant positive correlation between CTL score and neoantigen levels: melanoma, colorectal, endometrial, lung adenocarcinoma, and endocervical adenocarcinoma (Fig. 2f). Notably, these include the majority of cancer lineages well-studied in the context of immunotherapy, and are consistent with previous reports of mutational load predicting patient response to checkpoint blockade^3,5–7^.

### Cytokines are predictive of CTL infiltration across cancers

**Fig. 3:**
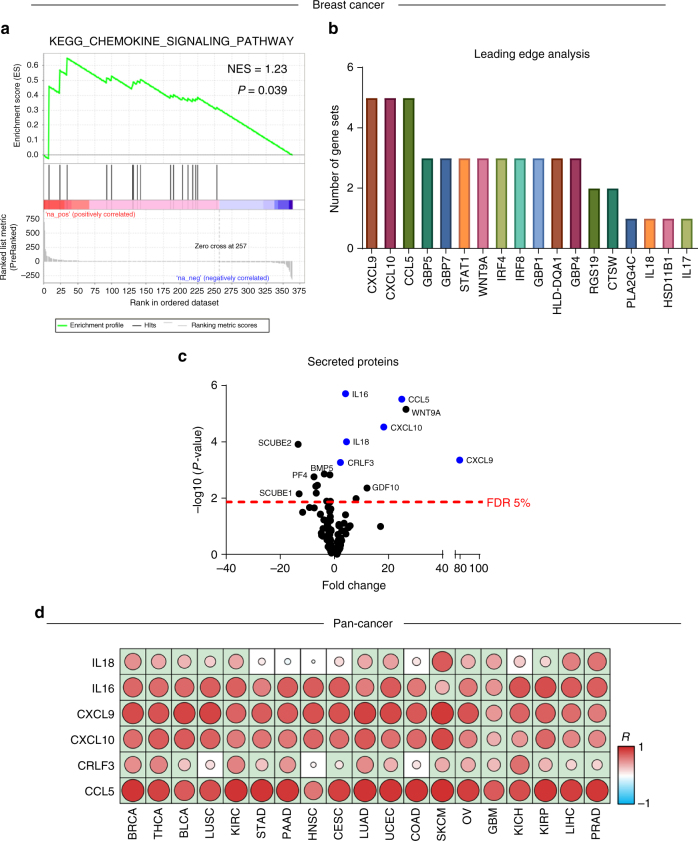
Identification of cytokines responsible for CTL invasion across multiple cancers. **a** Gene set enrichment analysis for total proteins in CTL high breast cancer.** b** Leading edge analysis of top ten enriched gene sets shows soluble factors are driving most of the enriched gene sets. **c** Volcano plot showing differential expression of secreted proteins in CTL high vs. CTL low patients. Dotted line indicates false discovery rate (FDR) of 5%. Proteins which show positive correlation between protein and gene transcript levels are indicated in blue. See Supplementary Fig. 4. **d** Pan-cancer analysis of correlation between CTL score and cytokine expression levels. Correlation coefficient (*R*) indicated by color of the spot, with significant correlations indicated by light green background. Sample sizes and exact *P*-values given in Supplementary Table 3

Next, in order to determine if there was a more global marker of CTL invasion, we performed gene set enrichment analysis (GSEA)^15^ on differentially expressed proteins between CTL high and low patients. Amongst the top hits was the “KEGG chemokine signaling pathway” (Fig. 3a). Leading edge analysis further identified multiple chemokines as potential drivers of the enriched phenotypes (Fig. 3b). Comparing the protein level of soluble factors in CTL high patients compared to CTL low patients we identified nine soluble factors that were over-expressed in CTL high patients at the protein level (Fig. 3c). In order to expand this across, cancers that do not have whole-proteome expression data available, we determined which soluble factors showed significant positive correlation between their transcript and protein levels, resulting in six candidate soluble factors (Supplementary Fig. 4). The expression level of these genes was correlated across 13 cancers using the RNAseq-based CTL score. Of these six genes, we found four to be significantly correlated with CTL levels across nearly all cancer lineages: *CXCL9, CXCL10, CCL5*, and *IL16* (Fig. 3d).

### Breast cancer CTL levels corresponds with ATM activation

**Fig. 4:**
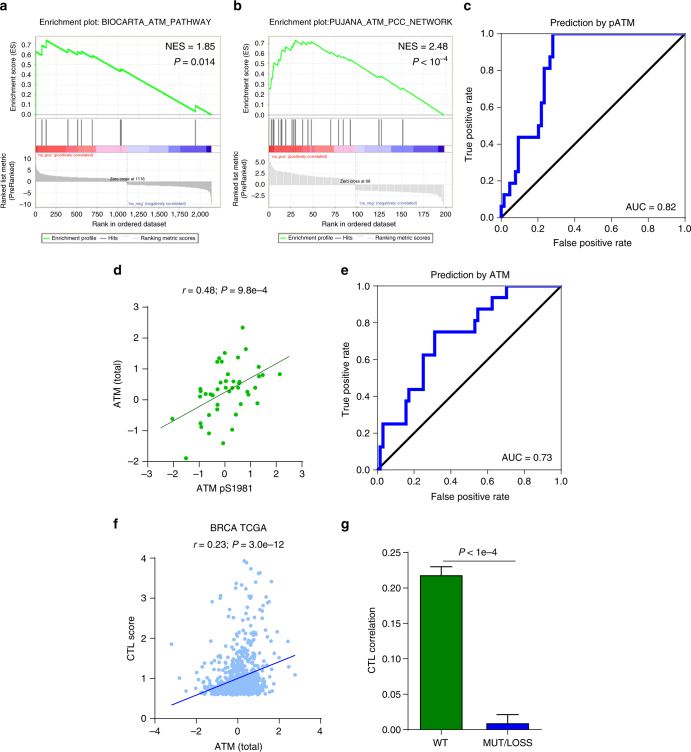
ATM as a driver of CTL invasion in breast cancer. **a** Gene set enrichment analysis for differentially expressed phospho-proteins shows enrichment of the ATM pathway. **b** Gene set enrichment analysis for breast cancer CTL high gene signature (see Supplementary Fig. 5) shows enrichment of ATM related genes. **c** Receiver-operator characteristic (ROC) plot for prediction of CTL high patients based on ATM phospho-S1981 levels in breast cancer patients. Area under the curve (AUC) equal to 0.82 (*N* = 80). **d** Correlation of total ATM with ATM phosphorylated at serine 1981 (*N* = 80). Spearman correlation coefficient (*r*) and corresponding *P*-value indicated above plot. **e** ROC plot for prediction of CTL high patients by total ATM protein (*N* = 80).** f** Correlation of CTL Score with total ATM determined by RPPA for the TCGA breast cancer cohort (*N* = 892). Spearman correlation coefficient (r) and corresponding *P*-value is indicated in the above plot.** g** Analysis of breast cancer patients with genomic alteration of ATM shows loss of correlation between ATM and CTL invasion compared to patients with wild type (WT) ATM. Correlation coefficient distributions were determined by random subsampling of each population to ensure equal sample size. Error bar represents 95% confidence interval, *P*-values determined by Wilcoxon rank-sum test

To better understand the upstream pathways that may be leading to CTL invasion as well as cytokine secretion, we began by repeating the GSEA between CTL high and low patients for differentially expressed phospho-proteins, and found enrichment of the BioCarta ATM pathway (Fig. 4a). Ataxia-telangiectasia mutated (ATM) is activated in response to DNA double-stranded breaks where it acts as a signal transducer to primarily activate the DNA damage checkpoint^16^. Enrichment of ATM related genes was also found performing the same analysis with a gene expression signature indicative of CTL invasion (Fig. 4b, Supplementary Fig. 5). Consistent with these observations, phosphorylated ATM was strongly predictive of CTL invasion (Fig. 4c). While information on ATM phosphorylation is not broadly available within the TCGA, all samples profiled by reverse phase protein array (RPPA) have total ATM levels which correlate well with phosphorylated ATM levels (Fig. 4d). These total ATM levels from RPPA show good agreement with Western blot quantification (Supplementary Fig. 6), and also offer good predictive power (Fig. 4e). Leveraging these RPPA ATM protein levels, we expanded our analysis to all TCGA breast cancer patients, and found ATM levels remained strongly predictive of CTL score (Fig. 4f).

To delineate if the increased ATM was merely correlational or may play some functional role, we analyzed the correlation between ATM and CTL score in patients with wild type ATM compared to those with mutation or deletion of ATM. We hypothesized that if ATM were to play a functional role, the relationship would not be observed in patients with genetic ATM inactivation. We found that patients harboring genetic ATM inactivation showed no correlation between ATM protein levels and CTL score, suggesting that ATM may in fact play a functional role in the recruitment of CTLs (Fig. 4g).

### Activation of ATM as a driver of cytokine expression

**Fig. 5:**
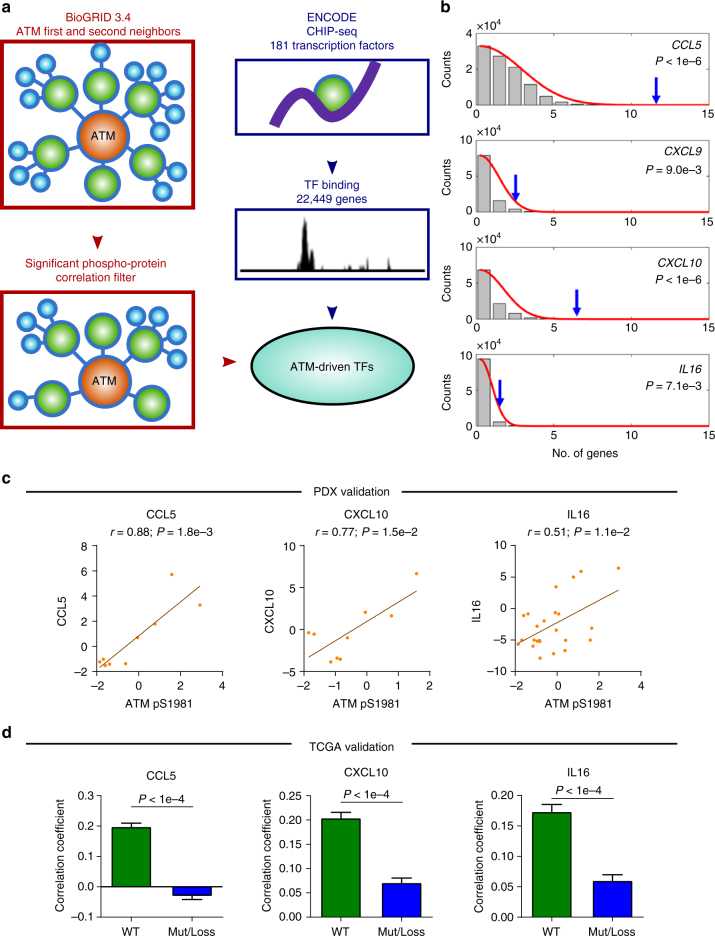
Enrichment of transcription factors for CTL cytokines in the ATM network. **a** Work flow to identify transcription factors within the ATM second neighbor network. **b** Enrichment of transcription factors that drive cytokines implicated in CTL recruitment. Blue arrows indicate number of transcription factors in ATM second neighbor network. The null distribution shown in the background represents number of transcription factors bound in randomly selected gene sets of equal size, which was used to determine an empirical *P*-value (inset). **c** Correlation of ATM phosphorylated at S1981 and cytokine protein levels in PDX breast cancer models (*N* = 27). Spearman correlation coefficient (r) and corresponding *P*-value indicated above plot. Error bars represent mean ± SEM.** d** Analysis of breast cancer patients with genomic alteration of ATM shows loss of correlation between ATM and cytokine expression compared to patients with wild type (WT) ATM. Correlation coefficient distributions were determined by random subsampling of each population to ensure equal sample size. Error bar represents 95% confidence interval, *P*-values determined by Wilcoxon rank-sum test. Error bars represent mean ± SEM

As we found cytokines to be potential drivers of CTL invasion, we hypothesized that ATM could be acting to increase their gene expression and subsequently recruit more CTLs. To test this, we integrated three primary data sets as shown in Fig. 5a. First, we created a network consisting of all first and second neighbor connections of ATM using curated protein–protein interactions from BioGrid 3.4^17^. Since ATM is a serine/threonine protein kinase, we then filtered these curated interactions to only include those that showed a significant correlation at the phospho-protein level. Finally, we recovered transcription factor binding information from ENCODE (ENCyclopedia Of DNA Elements) Project^18^ and tested to see if transcription factors bound at cytokine promoters were enriched within our ATM network. We found that all four primary cytokines correlated with CTL invasion were significantly enriched within this network (Fig. 5b). Analyzing this relationship in proteomic data from patient-derived xenografts (PDX) from immunocompromised NOD/SCID mice that lack mature T cells, B cells, and NK cells, we found that all three detectable cytokines (CCL5, CXCL10, and IL16) positively correlated with levels of phosphorylated ATM (Fig. 5c), suggesting these factors are tumor-cell derived^19^. We further validated these results in patient samples with the approach from Fig. 4g, where if the relationship is causal then the correlation between ATM levels and cytokine gene expression should be lost in patients with genetic ATM inactivation. Consistent with a potential causal role of ATM, all three cytokines tested showed significantly decreased correlation in tumors with genetic alterations in ATM (Fig. 5d).

**Fig. 6:**
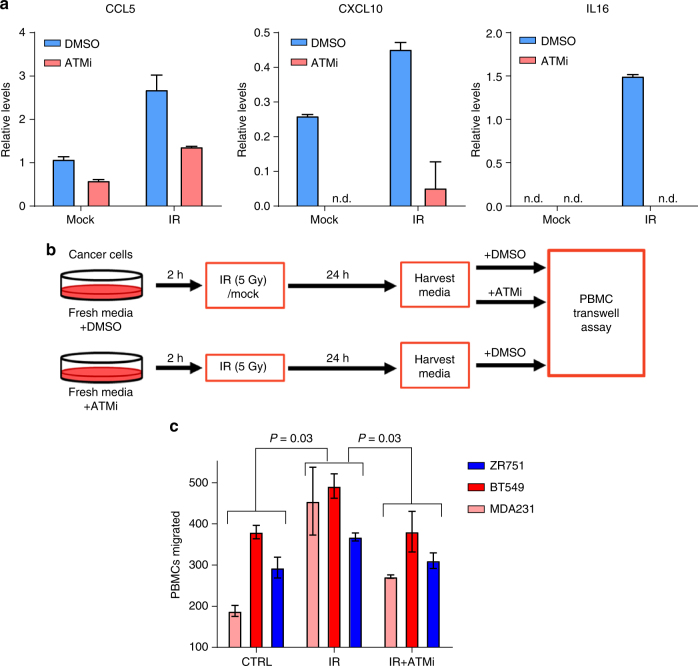
Phosphorylation of ATM induces cytokine secretion and PBMC migration. **a** Quantification of CCL5, CXCL10, and IL16 secretion by ELISA following irradiation of BT549 breast cancer cells either in presence or absence of ATM inhibitor KU-55933 at 10 μM. Values plotted as mean ± std of duplicate spots. See Supplementary Fig. 7.** b** Schematic for collection of conditioned media for PBMC transwells. Final concentration of ATM inhibitor KU-55933 was equal in all conditions (10 μM). **c** Migration of PBMCs towards tumor cell conditioned media from triple negative breast cancer cell lines MDA-MB-231 and BT-549 and luminal breast cancer cell lines ZR-75-1. Error bars represent ± standard deviation of duplicate runs. Significance determined by ANOVA using a Holm-Sidak post-hoc test

To validate this causal role for ATM phosphorylation in cytokine secretion, we next turned to in vitro culture models to directly manipulate phosphorylated ATM levels. First, ATM phosphorylation was induced by irradiation in breast cancer cells in presence or absence of the ATM inhibitor KU-55933. Twenty four hours later, culture media was harvested and probed for cytokine amounts using an ELISA array. These studies revealed that not only did ATM activation by irradiation induce increases in all three key cytokines observed in the PDXs, but that this increase was blunted by ATM inhibition (Fig. 6a, Supplementary Fig. 7). The role of this cytokine secretion in immune cell recruitment was then evaluated by recapitulating this experiment and testing the ability of peripheral blood mononuclear cells (PBMCs) to migrate to conditioned media through a porous transwell (Fig. 6b). As observed with cytokine secretion, irradiation-induced ATM phosphorylation increased the migration of PBMCs, which was blocked by treatment of the tumor cells with an ATM inhibitor (Fig. 6c).

**Fig. 7:**
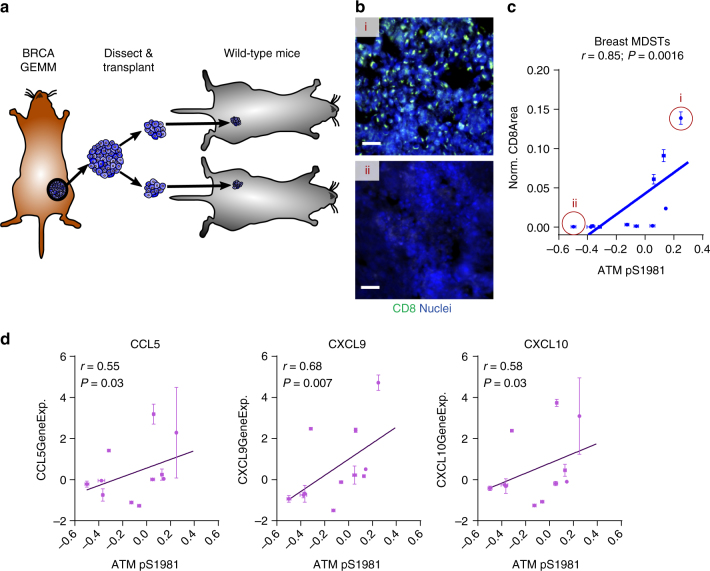
ATM phosphorylation correlates with CTL invasion and cytokine expression in a murine breast cancer model. **a** Schematic of murine derived syngeneic transplant (MDST) model. **b** Representative CD8 (green) immunostaining with DAPI nuclear counterstain (blue) of MDST tumor sections for a CTL-high MDST (i) and CTL-low MDST (ii). Scale bar = 50 μm. **c** Correlation of ATM pS1981 determined by RPPA with CD8 immunostaining (*N* = 11 MDST models). Normalized CD8 area was determined by taking the area staining positive for CD8 normalized to the entire area of the tissue section determined by autofluorescence. Models shown in **b** are indicated. Spearman correlation coefficient (r) and corresponding *P*-value indicated above plot. Error bars represent sample standard errors. **d** Correlation of ATM pS1981 determined by RPPA with gene expression values for *CCL5*, *CXCL9*, and *CXCL10* in the MDST model (*N* = 11 MDST models). Spearman correlation coefficient (r) and corresponding *P*-value indicated above plot. Error bars represent sample standard errors

Finally, to verify this phenotype we utilized a preclinical murine syngeneic transplantation breast cancer model (Fig. 7a)^14^. Within this model we found that phosphorylated ATM as quantified by RPPA showed significant positive correlation with CTL levels as determined by immunostaining for the marker CD8 (Fig. 7b, c). Moreover, phosphorylated ATM also positively correlated with expression of *CCL5*, *CXCL9*, and *CXCL10* (Fig. 7d). Taken together, these studies suggest that activation of ATM can act to drive cytokine expression and increase CTL recruitment to breast cancers.

### Neoantigens and ATM drive CTL invasion in distinct cancers

**Fig. 8:**
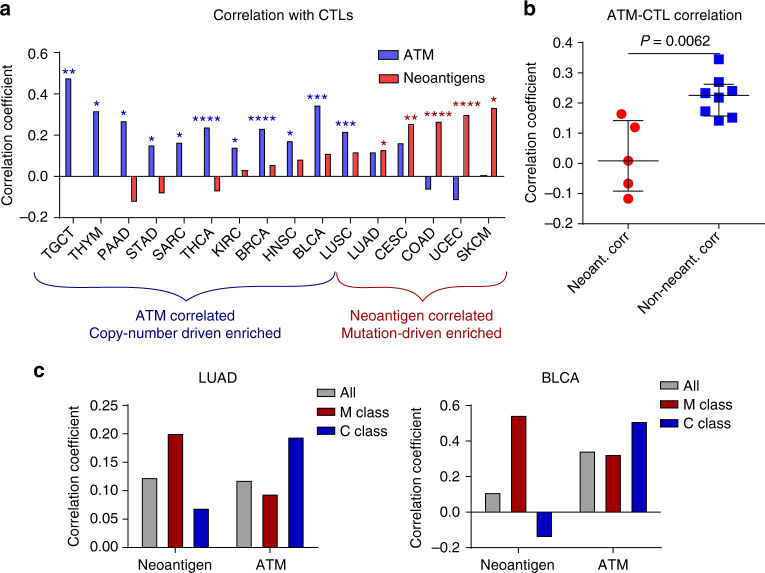
Neoantigen-independent CTL infiltration correlates with increased ATM across cancer lineages. **a** Spearman correlation coefficient between CTL score and total ATM determined by RPPA (blue bars), as well as between CTL score and neoantigen levels (red bars). Individual plots shown in Supplementary Fig. 8A. *P*-values determined from Spearman correlation coefficient and adjusted by Holm-Sidak method. *P < 0.05, **P < 0.01, ***P < 0.001, ****P < 1 × 10^-4^. **b** Correlation coefficient of total ATM protein and CTL levels. Cancers that had significant positive correlation with neoantigen levels showed a lower ATM/CTL correlation than those that did not (Non-neoant. Corr). P-value determined by Wilcoxon rank-sum test. Lines represent median and interquartile range. **c** Determination of correlation coefficients in M-class and C-class subsets. Correlation coefficients between CTLs and neoantigen load or ATM protein level were assessed for the bulk population (All), as well as M-class and C-class subsets. See Supplementary Fig. 9

Thus far we had observed that neoantigen load agreed well with CTL invasion in colorectal cancer and phosphorylated ATM was a larger determinant in breast cancer, but it remained unclear how generalizable these findings are. Using total ATM levels from RPPA to approximate phosphorylated ATM (Fig. 4d), we analyzed how well ATM correlated with CTL score and compared this with neoantigen correlations across 13 different cancer types with sufficient matched data for RPPA, mutations, and RNAseq (Fig. 8a, Supplementary Fig. 8A). Strikingly, correlation coefficients for CTL scores with ATM protein levels and neoantigen loads showed a strong negative correlation (*R* = −0.68, *P* = 9.8e-3). As we previously observed with colorectal cancer, many of the commonly studied cancers in immuno-oncology such as melanoma, lung, and endometrial cancers showed strong positive correlation between CTL scores and neoantigen levels consistent with previous literature^20^. In contrast, the majority of the remaining cancer lineages mirrored our observations in breast cancer, with minimal relationship between CTL levels and neoantigen load. In these cancers, we again observed significant positive correlation of CTL score with ATM protein levels. These findings were largely maintained when using CIBERSORT to predict CTLs instead of our CTL RNAseq score (Supplementary Fig. 8B–C). Consistent with our observations in breast cancer, this relationship appeared to be functional, as genetic ATM inactivation abrogated the correlation between ATM protein levels and CTL score/cytokine gene expression in the gastric cancer cohort (Supplementary Fig. 9).

Across cancer lineages, we found that positive CTL score correlation with neoantigens and ATM protein levels to be largely mutually exclusive. Indeed, the correlation of ATM with CTL scores was significantly higher in cancers where neoantigen levels were not indicative of immune infiltration (Fig. 8b). Upon closer inspection of these divergent phenotypes, we observed that they largely divided into the two classes of cancer originally observed by Ciriello et. al.^20^ Their pan-cancer analysis clustered tumor samples based on a panel of approximately 500 significant functional events known to drive cancer development and growth (Supplementary Fig. 10A), resulting in an M-class cluster, characterized by recurrent mutational drivers, and a C-class cluster, characterized by recurrent copy number alterations. Cancer lineages that were predominately M-class tumors showed primarily neoantigen-dependent CTL scores, whereas lineages that were predominately for C-class tumors showed dependence on ATM. While tumors from most lineages showed strong segregation into one class, lung adenocarcinoma (LUAD) and bladder cancer (BLCA) both had significant subsets of patients in each class (Supplementary Fig. 10A). We leveraged this fact to test our hypothesis, analyzing the correlation of CTLs with neoantigens in only M-class and only C-class patients from these lineages (Fig. 8c). Consistent with our hypothesis, both lineages showed an increase in Spearman correlation coefficient between neoantigens and CTLs in M-class tumors relative to the bulk population, and a decrease in correlation coefficient for C-class tumors. Likewise, the correlation between ATM and neoantigens was increased in C-class tumors compared to the bulk or M-class subset. This trend was largely recapitulated across all analyzed cancers (Supplementary Fig. 10B). Taken together, our results suggest that phospho-ATM may be a primary mediator of CTL invasion in numerous C-class tumors such breast, pancreatic, and gastric cancers.

## Discussion

In this study, we took a multi-omics approach integrating data from mutations at the DNA level, alterations in gene expression combined with proteomic and phospho-proteomic data to better understand the potential mechanisms to leading to recruitment of CTLs to the tumor microenvironment. First, we found that the proposed relationship between CTLs and neoantigen levels only held for a subset of cancers. Notably, in the majority of these cancers tended to belong to the M class of cancers identified by integrated pan-cancer analysis of TCGA data^20^. In this work, Ciriello and colleagues used ~500 key functional events to cluster 12 cancer types, and found that they divided tumors into two primary groups: a M class characterized by recurrent mutations and a C class characterized by recurrent copy number alterations^20^. These “M class” cancers that we find show positive correlation between neoantigen levels and CTL infiltration include most cancers where immunotherapy has been most successfully implemented, including colorectal cancers, lung adenocarcinomas, and melanomas. Indeed, clinical data supports the hypothesis that neoantigen load is indicative of patient response to checkpoint blockade in these cancer lineages^3,5–7^. However, upon analyzing other cancers including those originating from the breast, pancreas, and stomach, we found this relationship no longer held. Consistent with the lack of correlation between CTLs and neoantigen load in clear cell renal cancer (KIRC), a recent clinical trial found that mutational load does not indicate response to immunotherapy in kidney cancer^10^. These cancers all largely originate from the C class of cancers, characterized by frequent copy number aberrations. These cancers have been much less successfully targeted with current immunotherapy strategies, so a better understanding of CTL recruitment to these tumors is urgently needed to advance the potential of these powerful therapies.

To this end, by analyzing whole transcriptome and proteome data sets we identified phosphorylation of the DNA double-strand break response protein ATM as a novel potential driver of CTL invasion in breast and other C-class cancers. Phosphorylated ATM offered strong predictive value both in the CPTAC breast cancer patient cohort (Fig. 4c), as well as in a pre-clinical murine syngeneic transplant model (Fig. 7b). Moreover, this correlation with ATM was abrogated when analyzing ATM mutant breast (Fig. 4g) or gastric (Supplementary Fig. 9A) cancer patients, suggesting ATM plays a functional role in CTL recruitment and is not merely correlational. As we found levels of the cytokines *CCL5, CXCL9, CXCL10*, and *IL16* to indicate high CTL score across multiple cancers, we hypothesized that ATM in its role as a signal transducer may be promoting the transcription of these cytokines. Indeed, ATM network modeling showed enrichment of transcription factors that bind to cytokine promoters (Fig. 5a, b). Moreover, we found that inducing ATM phosphorylation through irradiation elevated secretion of cytokines and promoted PBMC migration (Fig. 6a,c), both of which were reversible with ATM inhibition.

Overexpression of either *CCL5*^21^ or *CXCL10*^22^ can abrogate the in vivo tumorigenicity of cancer cell lines via increased immune cell recruitment without any alterations in in vitro growth kinetics. This raises the interesting proposition that radiation may be useful clinical modality to combine with immunotherapy independent from the proposed abscopal effect of antigen release. Pre-clinical studies in melanoma have shown that irradiation alone can increase CTL invasion and that an intact immune system is required for efficacy of radiotherapy even in absence of checkpoint inhibition^23^. Similar enhancement of CTL recruitment has been seen following irradiation in breast cancer studies^24^. A number of clinical trials combining these two therapies are currently underway, and this further mechanistic understanding of how they may synergize could improve aspects of study design including dose scheduling^25^. More broadly, it will also be of interest to determine alternative ways to induce immune response when radiotherapy is not an option or greatly limited. Recent studies have shown that PARP inhibitors may be useful for treating a subset of pancreatic cancer patients^26^, and that T-cell invasion is marker of good prognosis in this lethal cancer^27^. With accumulating evidence that PARP inhibitors may act synergistically with immunotherapy^28^, this could be an attractive option for targeting this aggressive disease.

As the immunotherapy field comes of age, it will remain critical to understand cancer-type specific influences of immune cell recruitment necessary for efficacy. While this study provides evidence of two divergent drivers between C- and M-class cancers, undoubtedly further specification exists within each class. Predictive biomarkers will be critical to moving forward clinical trials as response rates in unselected patients currently remain low, and mechanistic insight into the function of these biomarkers may be used to induce responses in a larger patient population. Taken together, this research advances this goal by using an integrative analysis approach to elucidate phosphorylated ATM as a novel driver of CTL recruitment to tumors.

## Methods

### Analysis of proteomic and phospho-proteomic data for detection of CTLs

Patients were considered CTL high in colorectal cancer if both CD8 and GZMB were detected, whereas CTL low was considered if neither protein was detected. Likewise, for breast positive expression of both CD8 and GZMB was determined as CTL high, whereas expression of neither protein was considered CTL low. To determine gene–protein correlations and differential gene expression, we interpreted absent proteins in the iTRAQ-labeled breast cancer data set on a per-run basis. We first determined if any samples within a given 4-plex detected the protein in question. If so, these samples were taken to be 10% the lowest detectable value, otherwise they were excluded from the analysis.

### CTL infiltration score from RNAseq gene expression

To determine CTL invasion from RNAseq expression data, we started with expression values for *CD8A*, *GZMB*, and *PRF1*, three markers previously shown to best predict CD8^+^ T-cells and cytolytic activity^13^. Expression values were transformed as log2(counts + 1). We performed elastic net regression with these against the breast cancer CTL high patients, resulting in a predictive signature consisting only of *CD8A* and *GZMB* (Supplementary Table 4). This method compared favorably with other previously published methods^4,29^ (Supplementary Fig. 3).

### Gene set enrichment analysis of CTL high tumors

Gene set enrichment analysis (GSEA) and leading edge quantification was performed as previously described^15^. For proteomic and phospho-proteomic analysis, proteins were ranked by their T statistic. For gene expression analysis, we adapted our previously published algorithm^30^ to develop a representative gene signature. In short, the breast cancer cohort was divided into a testing and a training group. Differential analysis was determined by sub-sampling the training group 1000 times to generate 1000 lists of *P* values and fold changes. Optimal *P*value, fold change, and the percentage of time these values must be satisfied was determined using a grid search algorithm resulting the final signature (Supplementary Fig. 5). The coefficients for this signature were used to run GSEA based on gene expression.

### Enrichment of cytokine transcription factors in ATM neighborhood

Protein–protein interactions were called from BioGrid 3.4^17^ to generate an initial network with all first and second neighbors of ATM. We then filtered these curated interactions to only include those which showed a significant correlation at the phospho-protein level (FDR of 10%) to generate a final ATM second neighbor network. Transcription factor binding information from ENCODE project was used to test if cytokine transcription factors were enriched within this network. To do so, we determined the number of cytokine TFs in the ATM network, and then compared this to an empirical null distribution created by randomly selecting proteins sets equal to the size of the ATM network for 10^6^ iterations.

### Collection of conditioned media and ELISA

RPMI containing 2% FBS was equilibrated overnight in an incubator at 5% CO_2_ and 37 °C. The following day, this media was added to sub-confluent BT-549, MDA-MB-231, or ZR-75-1 breast cancer cells (ATCC) with the addition of either 10 μM KU-55933 (Selleckchem, Houston, TX) or DMSO solvent control. Two hours later, cells were either irradiated (5 Gy) or mock treated. Conditioned media was collected 24 h later, centrifuged at 800 g for 10 min, and filtered through a 0.45 μm filter to remove any cells or debris. Following collection cells were counted, and media diluted to correct for any discrepancies. The Proteome Profiler Human Cytokine Array Kit was acquired from R&D Systems (Minneapolis, MN) and run essentially per manufacturer’s instructions. Spot intensity was quantified using ImageJ software.

### Peripheral blood mononuclear cell migration assay

Transwells (6.5 mm, 3.0 μm pore size) were acquired from Corning (Kennebunk, ME) and equilibrated overnight in RPMI containing 2% FBS. Cryopreserved peripheral blood mononuclear cells were acquired from ZenBio (Research Triangle Park, NC) and thawed per provided instructions. To account for any effects of ATM inhibition, KU-55933 was added directly to the PBMCs, and either KU-55933 or DMSO was added as required to cancer cell supernatants. Each transwell received 2 × 10^5^ PBMCs which were allowed to migrate for 4 h. After removing the transwells, the plates were centrifuged and migrated PBMCs counted using an IncuCyte Zoom (Essen Bioscience).

### Mouse models and immunofluorescent staining

Previously described murine-derived syngeneic transplant models (MDSTs) models were transplanted into 4–8 week old FVB/N mice^14^. Tumors were fixed in 10% formaldehyde, embedded in paraffin, and sectioned on a Finesse 325 microtome (Thermo Scientific) into 7 μm sections. Sections were deparaffinized with xylene and rehydrated through an ethanol gradient. Antigen retrieval was performed using 10 mM citric acid (pH 6.0) at 95 °C for 30 min. Sections were treated with 3 mg/mL sodium borohydride to reduce autofluorescence, blocked with normal goat serum, and incubated overnight at 4 °C in a humidified chamber with 0.5 μg/mL rat anti-CD8 (clone 4SM15, eBioscience). After washing sections were incubated with secondary antibody (cross-adsorbed goat anti-rat AlexaFluor 488, Invitrogen) for 1 h at room temperature. Slides were counterstained with Hoechst and mounted with Vectashield (Vector Labs). Images (at least eight random fields per section) were acquired on a Nikon Eclipse TI inverted microscope at ×20 magnification. Images were quantified in a custom-written MATLAB algorithm. Area staining positive for CD8 was segmented, and normalized to total tissue area determined from residual autofluorescence in the red channel.

### ATM mutation analysis

Gene expression values for *CD8A*, *GZMB*, *CCL5*, *CXCL10*, and *IL16*, as well as ATM protein levels (RPPA) and ATM mutation status were obtained from cBioPortal. We randomly selected a subset of ATM mutant and wild-type patients to calculate the correlation coefficient for ATM and desired gene levels. This process was repeated 1000 times, and a Wilcoxon rank-sum test was used to determine if the correlation was significantly altered.

### Data availability

Proteomic data was acquired from CPTAC publications for breast^11^ and colorectal cancer^12^ patients. Whole transcriptome data for breast cancer was downloaded from the GDC data commons (https://portal.gdc.cancer.gov/). Pan-cancer gene expression, RPPA data, and mutation data were retrieved from cBioPortal^31,32^. We also utilized omics data sets for breast cancer PDXs^19^ and MDST models^14^. Transcription factor binding information from ENCODE (ENCyclopedia Of DNA Elements) Project^18^ was downloaded from Harmonizome^33^. Neoantigen levels were retrieved from The Cancer Immunome Atlas^4^. Classification of tumors into M-Class and C-Class was obtained from Ciriello et al.^20^

## Electronic supplementary material

Supplementary Information(PDF 2406 kb)
