# Supplementary material for: Multi-omics analysis reveals neoantigen-independent immune cell infiltration in copy-number driven cancers
Source: Nat Commun. 2018 Apr 3;9:1317. doi: 10.1038/s41467-018-03730-x (PMC5882811; doi:10.1038/s41467-018-03730-x)
Supplement: Supplementary file 1 — Supplementary Information(PDF 2406 kb) [file 41467_2018_3730_MOESM1_ESM.pdf]

SUPPLEMENTARY INFORMATION FOR McGrail *et al.*

Multiomics analysis reveals neoantigen-independent immune cell infiltration in copy-number driven cancers

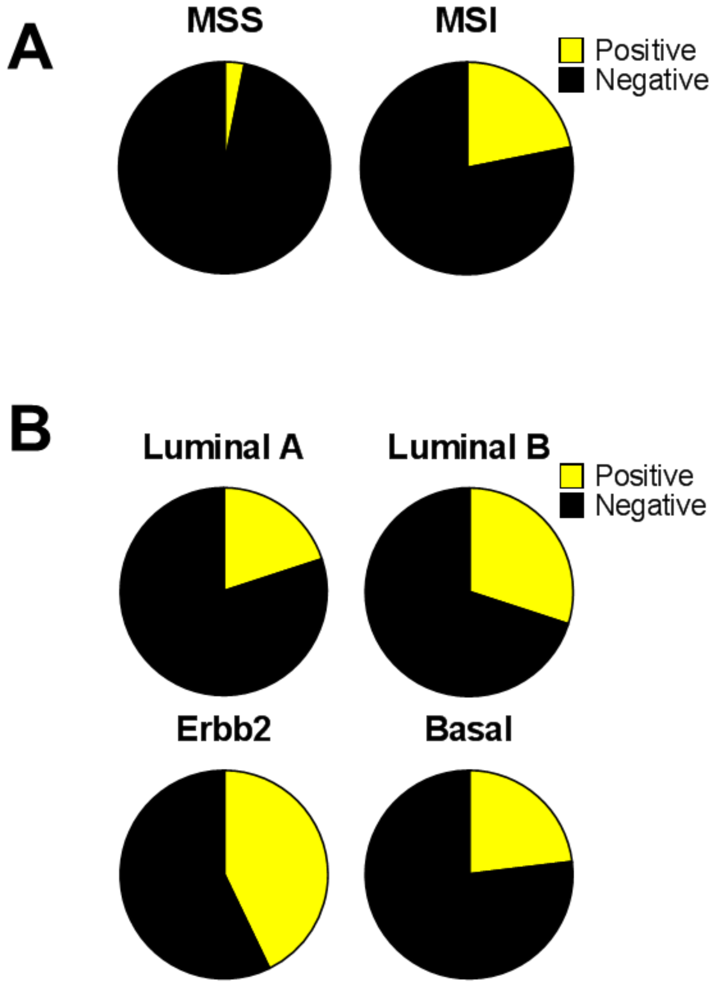

Supplementary Figure 1. CTL high patients by cancer subtype. (A) Fraction of microsatellite stable (MSS) and microsatellite instable (MSI) patients classified as CTL high in colorectal cancer. (B) CTL high breast cancer patients show no significant enrichment in any single subtype.

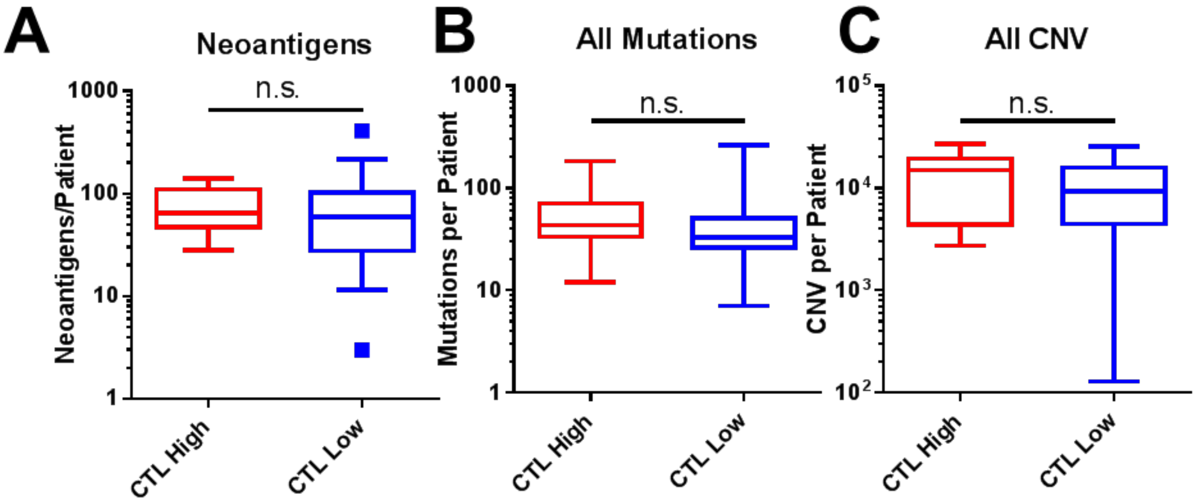

Supplementary Figure 2. Comparison in genomic alterations between CTL high (N=16) and CTL low (N = 44) breast cancer patients. (A) Number of neoantigens per patient in CTL high and low breast cancer patients. (B) Number of mutations per patient in CTL high and low breast cancer patients. (C) Copy number variations per patient in CTL high and low breast cancer patients. Box indicates median with interquartile range, and whisker length determined by the Tukey's method. The interquartile range (IQR) is defined as the difference between the 25th and 75th percentiles. The upper whisker marks the largest value less than the sum of the 75th percentile plus 1.5IQR, while the lower whisker marks the lowest value greater than the 25th percentile minus 1.5IQR.

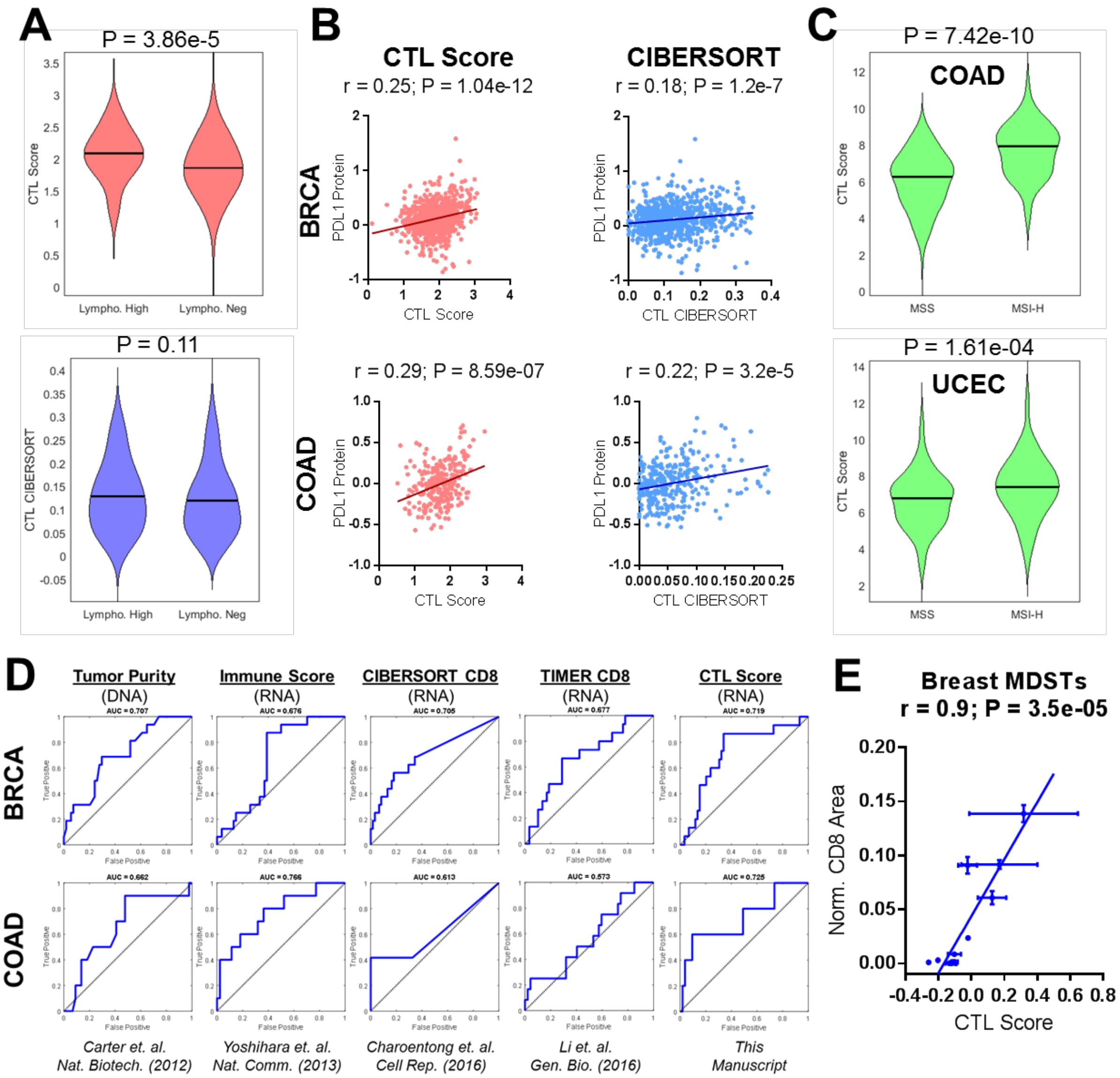

Supplementary Figure 3. Evaluation of RNAseq CTL score. (A) Breast cancer patients from the TCGA were divided based on lymphocyte infiltration levels as evaluated by histopathologists on tissue slides, showing enrichment of our RNAseq CTL Score (left) but not CIBERSORT CTLs (right). Significance determined by Wilcoxon rank-sum test. (B) Both CTL score and CTL levels determined by CIBERSORT show positive correlation with RPPA-determined PD-L1 protein levels in breast and colorectal cancer patients from the TCGA. (C) CTL score is increased in microsatellite high (MSI-H) cancer patients from the TCGA compared to microsatellite stable (MSS) patients for both colorectal (COAD) and endometrial (UCEC) cancers. (D) Receiver-operator characteristic (ROC) curves for specified predictors of immune infiltrate for breast (BRCA) and colorectal (COAD) cancer patients. Area under the curve (AUC) for each ROC curve shown above. (E) Comparison of CTL RNAseq score with area staining positive for CD8 in murine derived syngeneic transplant model, with each dot representing an individual syngeneic transplant

model. For CD8 stain quantification, the total area staining positive for CD8 was determined using automated image analysis and then normalized to total tissue area for each image. Data are plotted as mean +/- S.E.M. from at least two tissue sections and two RNAseq replicates. Pearson correlation coefficient (r) and p-value are given. N = 13.

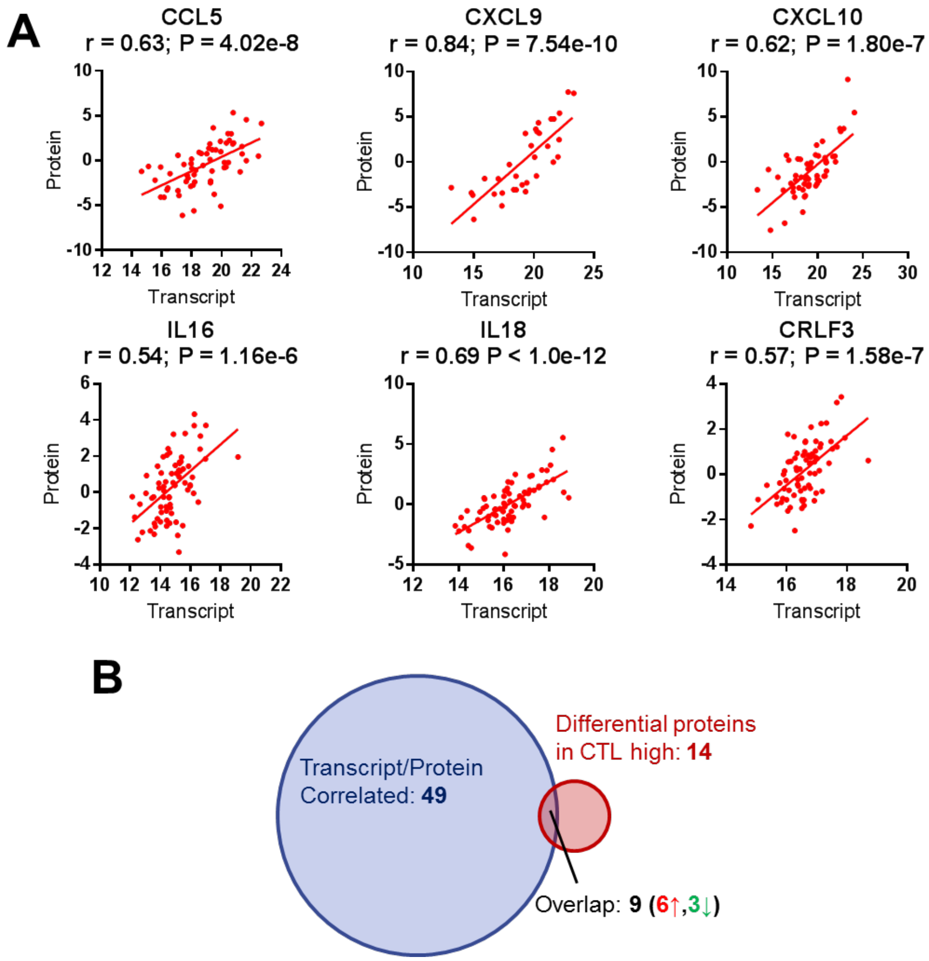

Supplementary Figure 4. Gene-protein correlation of up-regulated cytokines in CTL breast cancer patients. (A) Significantly up-regulated cytokines that also showed positive gene-transcript correlation (N = 74). (B) Venn diagram showing positively correlated transcript/protein levels for secreted factors, as well as secreted factors differentially regulated at the protein level. Soluble factor genes were taken from the "NABA_SECRETED_FACTORS" gene set.

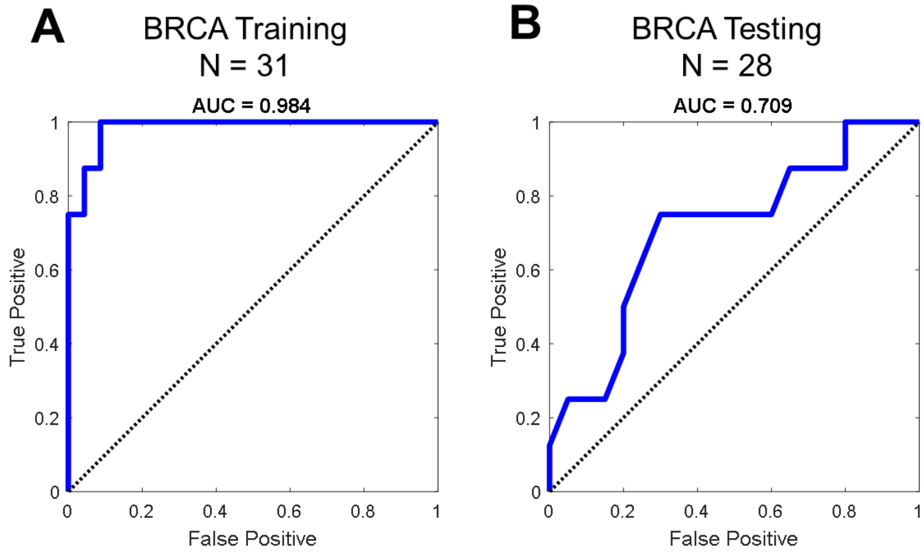

Supplementary Figure 5. Gene signature for CTL high patients in breast cancer. (A) Receiver-operator characteristic curve for training set. (B) Receiver-operator characteristic curve for testing set.

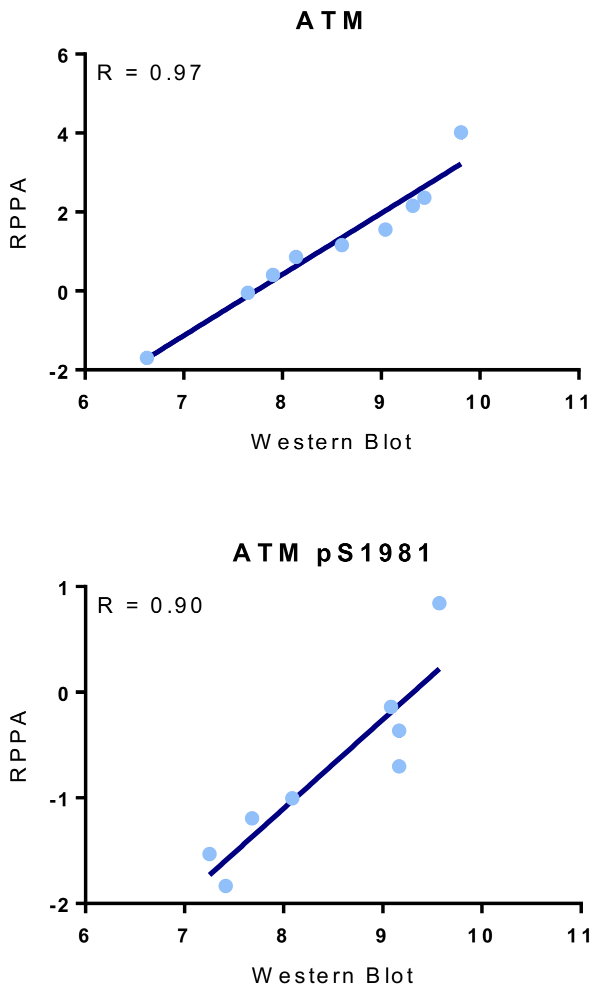

Supplementary Figure 6. Comparison of western blot to RPPA for ATM and phospho-ATM, related to Figure 4. Inset R indicates Pearson correlation coefficient.

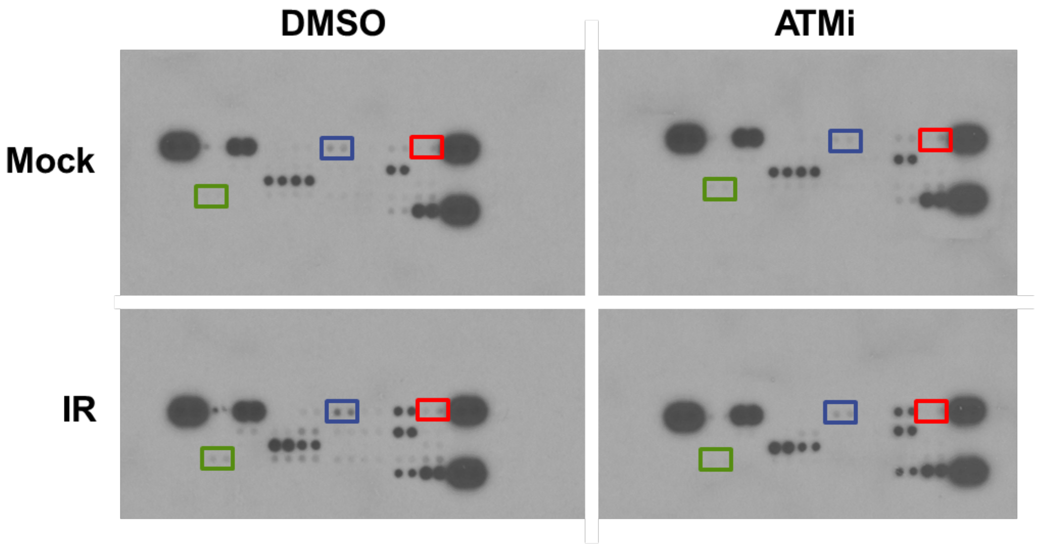

Supplementary Figure 7. Raw ELISA scans, related to Figure 6. Blue box indicates CCL5, red box indicates CXCL10, green box indicates IL16.

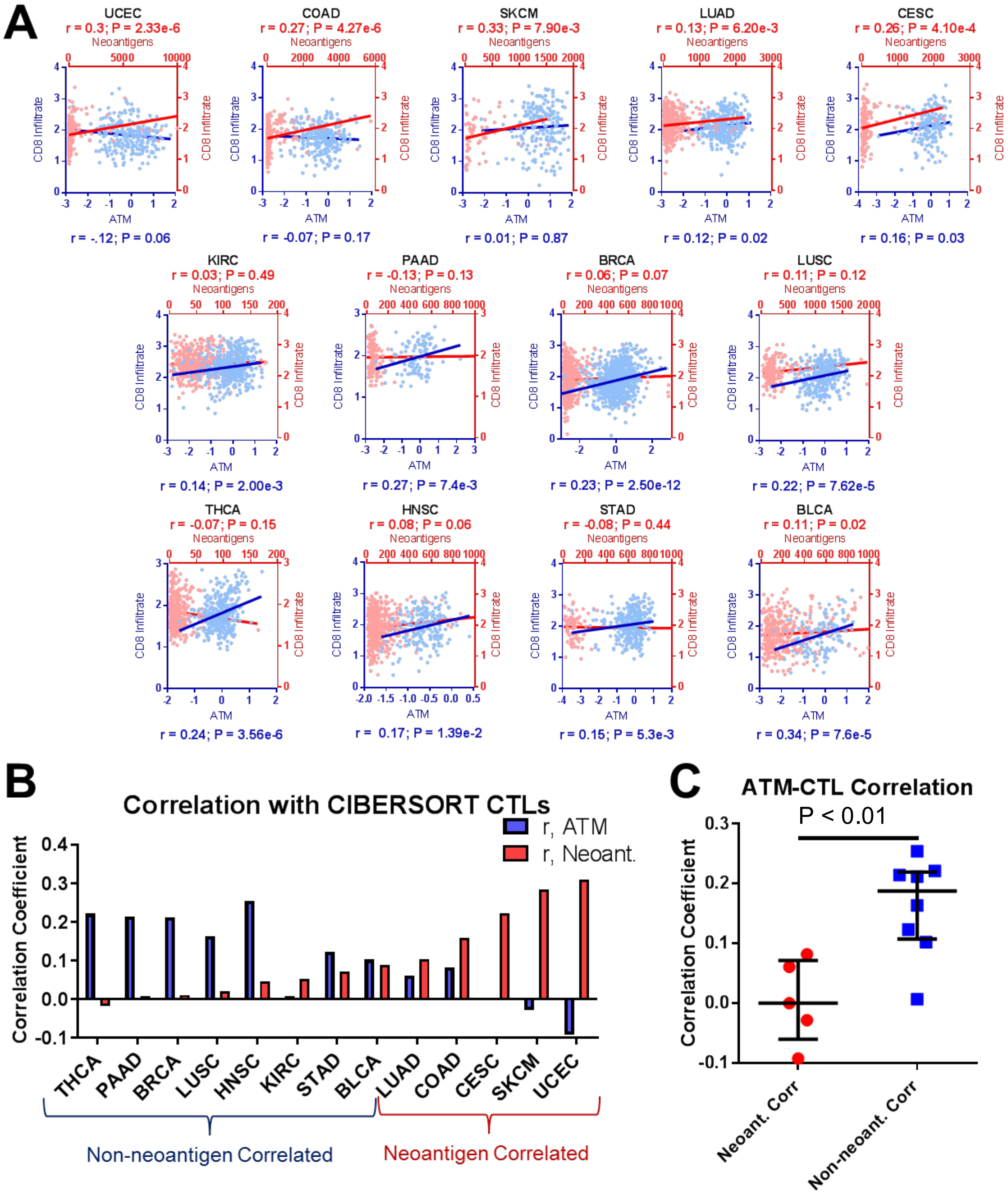

Supplementary Figure 8. Raw correlation plots and validation with CIBERSORT. (A) Individual plots of CTL score plotted against neoantigen levels (red) and ATM protein level (blue). Spearman correlation coefficient and p values shown. (B) Spearman correlation coefficients for between CIBERSORT CTL score and ATM (blue) or neoantigens (red). (C) Correlation between ATM and CTLs determined by CIBERSORT is significantly increased in cancer lineages with neoantigen-

independent CTL levels, exhibiting an identical Wilcoxon P-value to that obtained using our CTL score. Lines represent median and interquartile range.

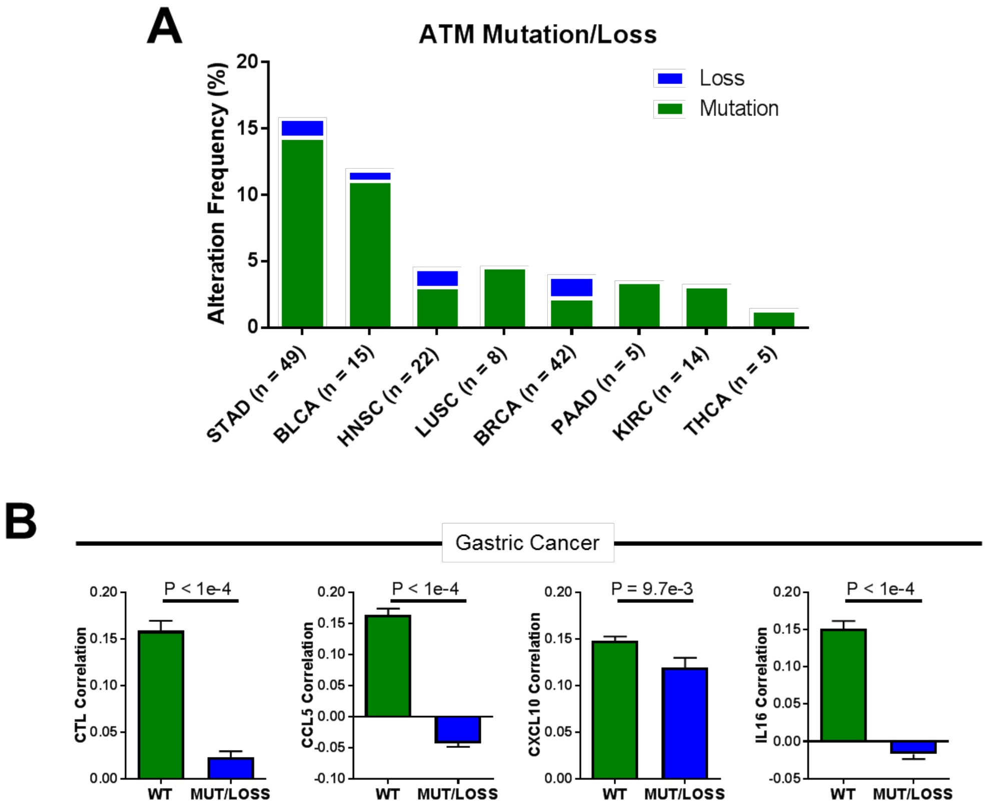

Supplementary Figure 9. Analysis of samples with genetic alterations in ATM. **(A)** ATM genetic alteration frequency in cancers with positive ATM/CTL score correlations. Inset n indicates total number of samples with alterations. Sample size in gastric (STAD) and breast (BRCA) were sufficient for analysis of genetically altered cohorts. **(B)** Analysis of breast cancer patients with genomic alteration of ATM shows loss of correlation between ATM and CTL invasion as well as cytokine expression compared to patients with wild type (WT) ATM. Correlation coefficient distributions were determined by random subsampling of each population to ensure equal sample size. Error bar represents 95% confidence interval, P-values determined by Wilcoxon rank-sum test.

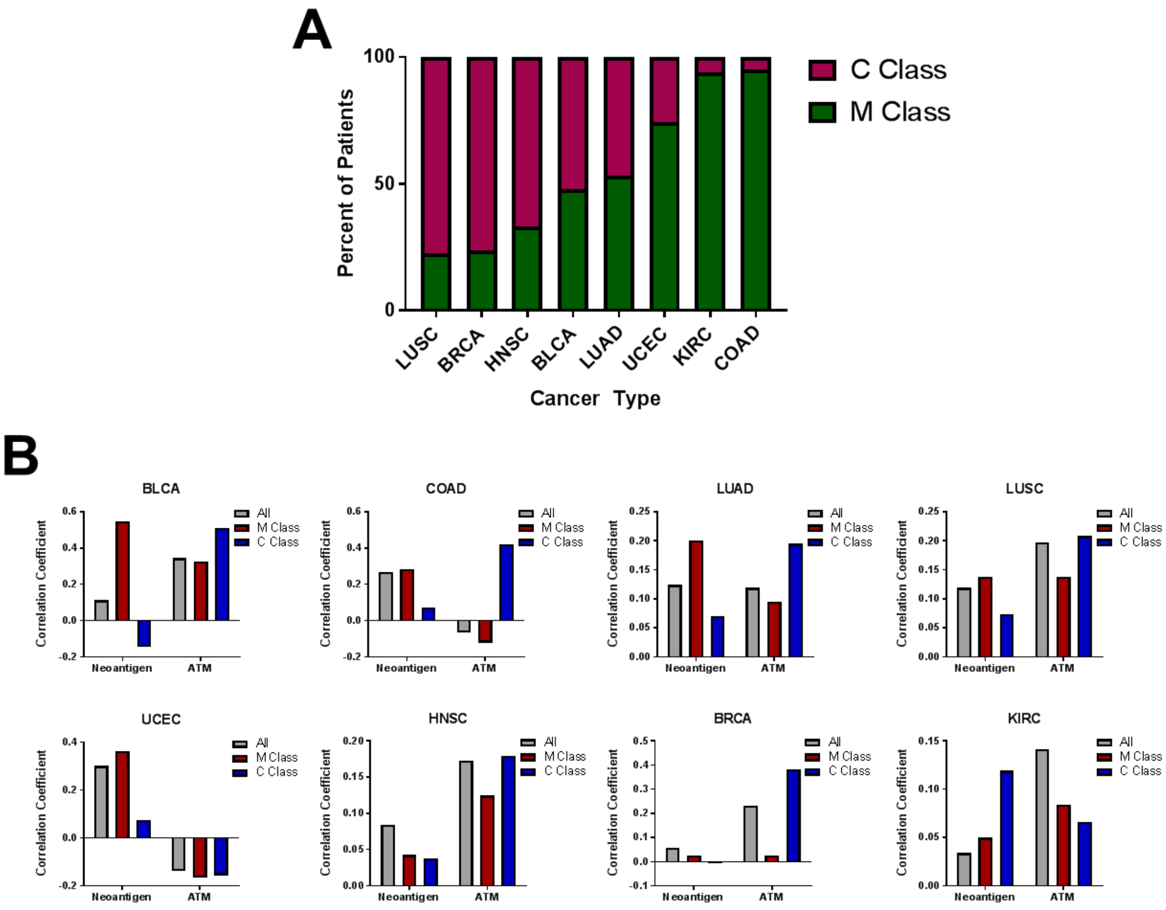

Supplementary Figure 10. Analysis of M-class and C-class tumors as defined by Ciriello et. al. **(A)** Percentage of tumors from each lineage that cluster into either the M-class characterized by recurrent mutational drivers or the C-class driven by recurrent copy number alterations. **(B)** Analysis of correlation between CTL levels and ATM protein/neoantigen load in bulk tumor population (All) as well as after division into M-class and C-class tumors for each lineage.

<u>Supplementary Table 1.</u> Sample sizes for Figure 2D-E

Cancer	N
KICH	66
GBM	145
LIHC	194
PRAD	420
BLCA	391
COAD	288
OV	186
THCA	376
KIRP	161
UCEC	238
BRCA	963
STAD	85
PAAD	145
HNSC	482
CESC	187
SKCM	341
LUAD	486
LUSC	176
KIRC	415

<u>Supplementary Table 2.</u> Sample sizes and P-values for Figure 2F

Cancer	P	N
UCEC	2.4E-06	238
COAD	4.5E-06	288
CESC	4.1E-04	187
KICH	0.25	66
LUAD	6.2E-03	486
LUSC	0.12	176
BLCA	0.03	391
HNSC	0.06	482
SKCM	0.25	341
BRCA	0.07	963
GBM	0.54	145
KIRC	0.49	415
KIRP	0.80	161
OV	0.51	186
PRAD	0.14	420
THCA	0.15	376
STAD	0.44	85
LIHC	0.21	194
PAAD	0.14	145

Supplementary Table 3. Sample sizes and P-values for Figure 3D

Cancer	N	CCL5	CRLF3	CXCL10	CXCL9	IL16	IL18
BRCA	120	<1e-15	8.1E-06	<1e-15	<1e-15	<1e-15	1.4E-08
THCA	120	<1e-15	2.5E-08	<1e-15	<1e-15	<1e-15	0.04
BLCA	120	<1e-15	1.2E-03	<1e-15	<1e-15	<1e-15	5.7E-05
LUSC	120	<1e-15	0.05	<1e-15	<1e-15	<1e-15	0.08
KIRC	120	<1e-15	0	<1e-15	<1e-15	<1e-15	3.5E-04
STAD	120	<1e-15	8.9E-07	<1e-15	<1e-15	<1e-15	0.37
PAAD	120	<1e-15	1.7E-07	<1e-15	<1e-15	<1e-15	0.32
HNSC	120	<1e-15	0.76	<1e-15	<1e-15	<1e-15	3.3E-03
CESC	120	<1e-15	0.03	<1e-15	<1e-15	<1e-15	0.14
LUAD	120	<1e-15	1.1E-05	<1e-15	<1e-15	<1e-15	7.9E-05
UCEC	120	<1e-15	6.9E-05	<1e-15	<1e-15	<1e-15	6.5E-07
COAD	120	<1e-15	3.0E-03	<1e-15	<1e-15	<1e-15	0.58
SKCM	120	<1e-15	5.7E-08	<1e-15	<1e-15	3.03E-05	<1e-15
OV	120	<1e-15	1.9E-05	<1e-15	<1e-15	<1e-15	1.0E-03
GBM	120	<1e-15	4.5E-05	7.76E-04	2.19E-06	1.27E-06	8.0E-06
KICH	120	<1e-15	3.1E-09	7.82E-05	<1e-15	<1e-15	0.05
KIRP	120	<1e-15	4.2E-05	<1e-15	<1e-15	<1e-15	9.4E-03
LIHC	120	<1e-15	1.2E-03	6.59E-13	<1e-15	<1e-15	<1e-15
PRAD	120	<1e-15	9.7E-04	<1e-15	<1e-15	<1e-15	<1e-15

<u>Supplementary Table 4.</u> Cytotoxic T-cell (CTL) Gene Signature

Gene	Coefficient
CD8A	0.213
PRF1	0.054
